# Supplementary material for: RNA-Seq Analysis Identifies Differentially Expressed Genes in the Longissimus dorsi of Wagyu and Chinese Red Steppe Cattle
Source: Int J Mol Sci. 2022 Dec 26;24(1):387. doi: 10.3390/ijms24010387 (PMC9820533; doi:10.3390/ijms24010387)
Supplement: Supplementary file 1 [file ijms-24-00387-s001.zip › ijms-2030540-supplementary.pdf]

Table S1 The details of RT-qPCR primers

Gene name	EnsenbleID	Sequences of primers
ASIP	NM_206843.2	5’-GGACATCAGGCACCCAGAA-3’<br>5’-TGAGGAAGCACAGGCAGAC-3’
GINS2	XM_002694751.6	5’-ACCTGAAGCAGAGACAGAAGT-3’<br>5’-GGAACTTGGCTATGCGAGTG-3’
FBP1	NM_001034447.2	5’-AAGGCAGGAGGAATGGCTAC-3’<br>5’-TCCAGGAACTCAGTCACATCTT-3’
PLCE1	NM_001205852.2	5’-TGGTGGCTGCGGTTATGT-3’<br>5’-CAGAGTATTGCGGTGGATGG-3’
SDC3	NM_001206531.2	5’-GCTGGACGACCTCTACTCTG-3’<br>5’-GCTCTTCTGGCACCTCTGT-3’
GMNN	NM_001025337.2	5’-CGGCTGGATCTCTAGTTGGAA-3’<br>5’-AATGCTTCTTGGCTGACTCCT-3’
SCARF2	XM_005218303.3	5’-GCTTCAGTCGTATCAGCATGA-3’<br>5’- GCCTTCGTCTGTGGTATCG-3’
DHRS3	NM_174180.3	5’-CCTCAGCCTTCGCCTTCAT-3’<br>5’-GGAACATCTCGGTGCTTGTG-3’
ANXA2	NM_174716.1	5’-CGCAGCAATGAACAGAGACA-3’<br>5’-TGGTTGGTCCTTGAGCAGAT-3’
MRVI1	NM_174392.4	5’-CTCTGCTTCTGTGCCAACTC-3’<br>5’-CTTCTCTGCTCCTTCTCCTCTT-3’
CD44	NM_174013.3	5’-CCTCGGATACCAGAGACTACG-3’<br>5’-CACACCTTCTCCTACTGTTGAC-3’
NUMBL	NM_001076834.1	5’-TGACAGCGACAGCATCAGT-3’<br>5’-ACTTCCTCCAGCCACCTCT-3’
ANKRD2	XM_015460708.1	5’-ACGGCACAATGGCAGACT-3’<br>5’-TCCTCCAACACCAGCATCTC-3’
ALDH9A1	NM_001046423.1	5’-AGAGCCAACGACACCACTT-3’<br>5’-TCTGCCGAATCCTGACTTCTT-3’
CRYAB	NM_174290.2	5’-AGAGGAACTCAAGGTCAAGGT-3’<br>5’-GGATGAAGTAATGGCGAGAGG-3’
EHHADH	NM_001075780.2	5’-CTCCTCTGTTGGCGTTCTC-3’<br>5’-GCTGCTTCTTGTCTGATTCTAC-3’
β-ACTIN	NM_173979.3	5’-AGAGCAAGAGAGGCATCC-3’<br>5’-TCGTTGTAGAAGGTGTGGT-3’

Table S2 Primers to clone gene CDS sequences

Gene name	Gene ID	Primer sequences
ANKRD2	XM_015460708.1	5’-GTGGAGAATTGGGCCAGTGA-3’<br>5’-GTGTATGCAGAGGAATGGTGTC-3’
		5’-GCTAGCATGGCAAAAGCACCTAGCG-3’<br>5’-ATCGATTTACTCGGCTGCCACAGGCT-3’
ALDH9A1	NM_001046423.1	5’-CATGAGCACTGGCACCTTC-3’<br>5’-TGAAGAGCATCACTGAACCATT-3’
		5’-GCTAGCATGAGCACTGGCACCTTCG-3’<br>5’-ATCGATTCAAAAAGCAGACTCCACGTCA-3’
CRYAB	NM_174290.2	5’-GCTAGCATGGATATCGCCATCCACCAC-3’<br>5’-ATCGATCTACTTCTTGGGGGCTGCAG-3’
EHHADH	NM_001075780.2	5’-GGGCAACTGAGCAAACATGG-3’<br>5’-CCTGGCATAAGAAGCACATTCC-3’
		5’-ACGCGTATGGCTGAGTTCACGCGG-3’<br>5’-GCGGCCGCTCACAGTTTACTGCTGGGGG-3’

Table S3 The unannotated genes of DEGs in *Longissimus Dorsi* between Wagyu and Chinese Red Steppes

Gene	EnsenbleID	WC	RC	Log2FC	FDR	Style
ENSBTAG00000023318	ENSBTAG00000023318	15.23	0.68	4.47	4.94E-05	up
ENSBTAG00000010958	ENSBTAG00000010958	16.61	2.71	2.61	0.006637	up
ENSBTAG00000043547	ENSBTAG00000043547	16.68	2.73	2.61	3.28E-04	up
ENSBTAG00000000269	ENSBTAG00000000269	53.39	88.92	-0.73	0.045349	down
ENSBTAG00000038258	ENSBTAG00000038258	6.44	20.59	-1.67	0.022351	down
ENSBTAG00000014765	ENSBTAG00000014765	9.84	27.39	-1.4	0.018674	down
ENSBTAG00000030340	ENSBTAG00000030340	39.05	15.73	1.31	0.017844	up
ENSBTAG00000031834	ENSBTAG00000031834	234.79	101.34	1.21	0.040386	up
ENSBTAG00000009818	ENSBTAG00000009818	49.02	21.58	1.18	0.00945	up
ENSBTAG00000037991	ENSBTAG00000037991	68.22	30.48	1.16	0.0064	up
ENSBTAG00000007807	ENSBTAG00000007807	600.35	289.36	1.05	1.47E-10	up
ENSBTAG00000037709	ENSBTAG00000037709	142.27	69.28	1.03	0.005175	up
ENSBTAG00000026882	ENSBTAG00000026882	71.04	36.04	0.97	5.66E-04	up
ENSBTAG00000001079	ENSBTAG00000001079	43.77	86.37	-0.98	5.70E-04	down
ENSBTAG00000038536	ENSBTAG00000038536	33.45	98.92	-1.56	4.49E-04	down
ENSBTAG00000047883	ENSBTAG00000047883	199.14	103.54	0.94	6.78E-08	up
ENSBTAG00000015258	ENSBTAG00000015258	1525.07	813.08	0.91	0.023215	up
ENSBTAG00000008789	ENSBTAG00000008789	238.97	132.23	0.85	1.54E-12	up
ENSBTAG00000022570	ENSBTAG00000022570	8.68	112.07	-3.68	3.45E-06	down
ENSBTAG000	ENSBTAG0000	491.83	293.15	0.74	0.0035	up

00047148	0047148				46	
ENSBTAG000 00031483	ENSBTAG0000 0031483	1223.2 7	2079.8 8	-0.76	8.58E- 13	down
ENSBTAG000 00021906	ENSBTAG0000 0021906	0	40.37	-20	0	down
